# Exploring the Association between Amyloid-β and Memory Markers for Alzheimer’s Disease in Cognitively Unimpaired Older Adults

**DOI:** 10.14283/jpad.2024.11

**Published:** 2024

**Authors:** M.A. Parra, Y. Gazes, C. Habeck, Y. Stern

**Affiliations:** 1 Department of Psychological Sciences and Health, University of Strathclyde, Glasgow, UK;; 2 Cognitive Neuroscience Division, Department of Neurology, Columbia University Vagelos College of Physicians and Surgeons, New York, NY, USA

**Keywords:** Visual short-term memory binding, aging, cognitive marker, Alzheimer’s disease, biomarkers

## Abstract

**BACKGROUND:**

Memory tests vary in their sensitivity for detection of pre-symptomatic Alzheimer’s disease (AD). The Visual Short-Term Memory Binding Test (VSTMBT) identifies AD-related performance deficits in older adults who are otherwise cognitively unimpaired.

**OBJECTIVE:**

We investigated the association of this psychometric measure with brain amyloidosis and atrophy.

**DESIGN:**

Cross-sectional mixed and correlational.

**SETTING:**

Cognitive Reserve Study from Columbia University.

**PARTICIPANTS:**

a sample of 39 cognitively unimpaired older adults (Age: M=65.3, SD=3.07) was obtained from the above study.

**MEASUREMENTS:**

Extensive neuropsychological and neuroimaging (MRI and amyloid-β PET) assessments were carried out.

**RESULTS:**

Performance on the VSTMBT allowed us to split the sample into Low Binding Cost (LBC, N=21) and High Binding Cost (HBC, N=18). Groups were matched according to age [p=0.702], years of education [0.071], and sex [p=0.291]. HBC’s performance was comparable to that seen in symptomatic AD. Groups only differed in their amyloid-β deposition on PET in regions of the right ventral stream linked to visual cognition and affected early in AD pathogenesis (lateral-occipital cortex, p = 0.008; fusiform gyrus, p = 0.017; and entorhinal cortex, p = 0.046). Other regions known to be linked to low-level visual integration function also revealed increased amyloid-β deposition in HBC.

**CONCLUSIONS:**

VSTMB deficits are associated with neuropathogenesis (i.e., amyloid-β deposition) in the earliest affected regions in pre-symptomatic AD. The VSTMB test holds potential for the identification of cognitively unimpaired older adults with very early AD pathogenesis and may thus be a useful tool for early intervention trials or other forms of clinical research.

## Introduction

Assessment of memory in people with suspected Alzheimer’s disease (AD) has long focused on episodic memory, being its associative forms the most commonly targeted (1). This practice stems from the shared view that early AD pathology affects the hippocampus, a medial temporal lobe region known to support the formation of episodic memory via associative representations (2). Our understanding of memory decline in AD has increased considerably since traditional memory tests used to aid its diagnosis were developed. A hypothetical model of memory decline in AD rooted in Braak’s pathological stages (3) suggests that hippocampal atrophy, resulting from the accumulation of neurofibrillary tangles, appears rather late in the disease continuum. There is first a sub-hippocampal stage during which, regions of the anterior temporal lobe network are targeted by the disease. Such regions (e.g., entorhinal and perirhinal cortex) are involved in context-free memory functions such as familiarity-based recognition. Regions of this network are affected by AD earlier than the hippocampus (4), spared in normal aging (5), and involved in context-free memory (3). Therefore, tests that tax the functional integrity of this network will more likely detect AD-related impairments in its preclinical stages. Recent evidence supports this notion. For example, Norton et al. (6) focused on the entorhinal cortex and inferior temporal lobe as the former is thought to be the first location of tau buildup in AD while the latter represents the best proxy of early tau spreading to neocortex. The authors hypothesize that deficits in Visual Short-Term Memory Binding (VSTMB) would be more likely related to tau deposition in such regions given the evidence confirming its early decline in preclinical AD. It is worth noting that such regions are those thought to underpin context-free memory functions (3, 7) of which VSTMB is a clear example.

VSTMB (8) is a cognitive function that supports the integration and temporary retention of object’s features such as shape and color into unified representations. The function does not rely on the integrity of the hippocampus (9), is affected by AD prior to its hippocampal stages (10), and has proved insensitive to normal aging (11, 12). The VSTMB task (VSTMBT) is seemingly indexing very early neuropathological changes associated to the AD continuum. For instance, VSTMB correlates with Amyloid-β (Aβ) deposits in individuals who are in the preclinical stages of familial AD (i.e., E280A-PSEN1 mutation (6)) and in those expressing the early prodromal stages of sporadic AD (13) before any overt neurodegeneration is observed. The ability of memory tests to index AD pathology is a topic of ongoing research (14). Within the context, tests that assess the ability to hold bindings of items with own identity in memory (i.e., associative memory tests such as the Selective Reminding Test, FCSRT (15, 16)) or bindings of distinct features (shape, colors) which make up objects’ identity (i.e., conjunctive memory tests), have become increasingly popular (1). Accrued evidence suggests that conjunctive and associative forms of memory binding are dissociable (9, 17) with the former being supported by regions of the anterior medial temporal lobe network and the latter by regions of the posterior medial temporal lobe network (3, 7, 18). Although both forms of memory are affected in the early stages of AD, recent evidence suggests that the temporal pattern (i.e., “when”) of such impairments can now be detected with sensitive neuropsychological tests (19–21).

There is an urgent need for cognitive tests that can help detect the transition from normal to abnormal aging and monitor disease progression. Meeting such needs is proving challenging. Based on traditional neuropsychological and clinical assessments we have been allocating older adults who do not provide signals of AD (or other dementias) to control groups. Evidence has accrued suggesting that older adults who are still asymptomatic may be accumulating AD-related pathology and some show significant resilience to such changes (22). It will be ideal to identify memory tests which (1) are sensitive and specific to AD, (2) correlate with the accumulation of abnormal proteins in the brain linked to the development of AD dementia, and (3) are not sensitive to the brain changes that accompany normal aging. The VSTMB test seems to hold these properties. However, such a test has never been used to investigate if among those still healthy older adults there are individuals who show VSTMB decline that can be accounted for by the accumulation of AD related brain pathology. This was the aim of the present study. Based on the above reviewed evidence we predicted that cognitively unimpaired older adults with selective VSTMB impairment would also display a significant increase of Amyloid-β in their brains.

## Materials and Methods

### Participants

A cross-sectional mixed and correlational design was used. Participants were recruited primarily by randomized market mailing. An initial telephone screening determined whether participants met basic inclusion criteria (i.e., right-handed, English speaking, no psychiatric or neurological disorders, and normal or corrected-to-normal vision). Potentially eligible participants were further screened in person with structured medical and neuropsychological evaluations to ensure that they had no neurological or psychiatric conditions, cognitive impairment, or contraindication for MRI scanning. Global cognitive functioning was assessed with the Mattis Dementia Rating Scale, on which a minimum score of 130 was required for retention in the study. In addition, participants who met diagnostic criteria for Mild Cognitive Impairment (MCI) were excluded. A group of 39 healthy older adults [Age: 65.38 (3.06); Education in years: 16 (2.01); Gender M/F: 23/17] entered the study. The studies were approved by the Internal Review Board of the College of Physicians and Surgeons of Columbia University.

### Assessments

#### Neuropsychological test battery

Neuropsychological tests included tests of premorbid IQ (Wechsler Test of Adult Reading (WTAR (23)), WAIS-III Vocabulary subtest (24), memory and learning (Selective Reminding Test (25)), processing speed (Trail Making Test Part A (26), WAIS-III Digit Symbol subtest (24)), executive functions (Stroop Test (27), Trail Making Test Part B (26)), revision and monitoring (WAIS-III Letter-Number Sequencing (24)), and language (Category Fluency Test – Animals (28)) (See Supplementary Table 1 for the Battery used by (29) and the tests used in the present study).

#### The Visual Short-Term Memory Binding Task (VSTMBT)

**Figure 1.**
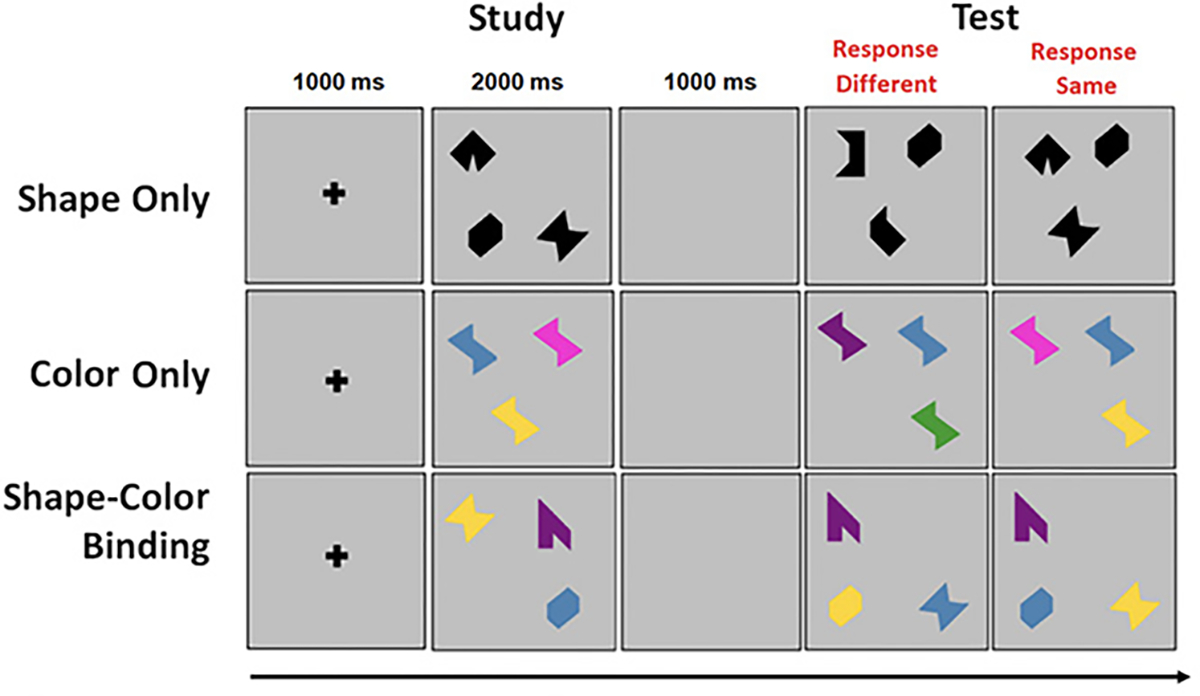
An example trial for each condition of the VSTMBT

The VSTMBT presented visual arrays of three stimuli each on a flat screen controlled by a PC. At the beginning of each trial, a fixation screen was presented for 500 msec. This was followed by the study display presented for 2000 msec. After a blank retention interval of 900 msec, the test display was presented. On 50% of trials, the study and test displays were identical. On the other 50%, there were changes between the study and test display. The task for the participant was to detect when a change had occurred and to respond orally ‘same’ or ‘different’ as appropriate. Items randomly changed locations across study and test display to avoid the use of location as a memory cue. There was then a gap of 1000 msec until the next trial (Figure 1).

The Shape only and Color Only conditions assessed VSTM for single features. The study arrays consisted of black shapes or colors (Figure 1). In the test display for the different trials, two shapes or colors from the study array were replaced by new shapes or new colors. In the shape-color binding condition, the arrays consisted of combinations of shapes and colors. In the test display for different trials, two shapes swapped the colors in which they had been shown in the study display. For each condition there was a practice session using flashcards. This was followed by 16 test trials. Trials were fully randomized across participants and conditions were blocked and delivered in a counterbalanced order. Participants had to pass a perceptual screening test in order to perform the VSTM binding task (30).

#### MRI and PET assessment

18F-florbetaben was donated by Piramal (Piramal Pharma, Inc.) PET scans were acquired on a Siemens Biograph64 mCT/PET scanner in dynamic, 3D imaging mode beginning 50 min after injection. Brain images were acquired in 4 X 5-minute frames over a period of 20 minutes. The images were immediately assessed for technical validity. If considered inadequate, the participant had an additional 20 minutes of continuous imaging. Transmission scans were done prior to the scan. If there was a repeat scan, transmission was done after the scan. We used a recently published, state-of-the-art automatic quantification technique to reconstruct Amyloid PET scans. The process started by aligning four dynamic PET frames to the first frame using rigid-body registration and generating a static PET image by averaging the four registered frames. The static PET volume was then registered with the CT and merged to generate a composite image. Each individual’s structural T1 scan, after being reconstructed with FreeSurfer, was registered directly to the static Amyloid PET volume using an inter-modal and intra-subject registration technique (rigid-body registration: 6 degree of freedom, mutual information). FreeSurfer regional masks were then used to extract regional uptake values. Regional and voxelwise amyloid SUVRs were calculated by dividing the regional and voxelwise uptake value by the average uptake value in the cerebellum gray matter region.

#### Magnetic Resonance Imaging data

All MR images were acquired on a 3.0T Philips Achieva Magnet. A T1-MRI sequence was recorded which was reviewed by a neuroradiologist. This sequence allowed us to obtain T1-weighted MPRAGE from which we derived measures of cortical thickness, with a TE/TR of 3/6.5 ms and Flip Angle of 8°, in-plane resolution of 256 × 256, field of view of 25.4 × 25.4 cm, and 165–180 slices in axial direction with slice-thickness/gap of 1/0 mm. Incidental findings were discussed with the subject’s GP and participants were informed that this would preclude further participation in the study. No participant in the current study had any significant MRI findings. FreeSurfer v5.1 (http://surfer.nmr.mgh.harvard.edu/) enable the reconstruction of individual T1 scans though which boundaries between white matter, gray matter and cerebral spinal fluid boundaries were visually inspected slice by slice. Manual corrections were undertaken when discrepancies were found until we reached satisfactory subject-level results. This analysis yielded mean cortical thickness for each participant which we then used for group-level analyses.

#### Statistical Analysis

##### Behavioral data

Based on previous studies involving older adults with and without cognitive impairment (12, 31, 32), we defined a cut-off score for the Cost of Binding. These earlier studies confirm that a drop of performance on the Shape-Color Binding condition relative to the single feature conditions above 20% is highly indicative of Alzheimer’s disease or risk of developing it (10, 21, 30). For instance, using a less cognitively demanding version of the VSTMBT that the version used in the current study, Della Sala et al. (32) showed that the Cost of Binding in patients with mild Alzheimer’s disease dementia was 23.3% while in Healthy Controls is was 2.9%, achieving an Area Under the Curve (AUC) of 97%. More recently, Forno et al. (21) confirmed that the cost of binding can help identify individuals in the Subjective Cognitive Decline stages who present with VSTMB deficits akin to those seen in AD patients. We decided to obtain the Cost of Binding for each individual participant using the Percentage of Correct Recognition drawn from each condition of the VSTMBT.


BindingCost=(AverageofScoreonSingleFeatures)–ScoreonShape-ColorBinding

The Average Score on Single Features was the average of the Percentage of Correct Recognition drawn from the Shape Only and Color Only conditions. The Score on Shape-Color Binding was the Percentage of Correct Recognition from the Shape-Color Binding condition.

Cognitively unimpaired older adults whose cost was greater than 20% were allocated to the group High Binding Cost (HBC) while those equal or below the cutoff were allocated to the Low Binding Cost (LBC) group. Based on the evidence reviewed in the Introduction, we predicted that HBC were those cognitively unimpaired older adults who would be significantly accumulating neuropathology in their brains.

For the analysis of data drawn from the VSTMBT, we used a mixed model with Group (LBC vs HBC) as the between-subjects factor and Condition (Color Only vs Shape Only, vs Shape-Color Binding) as the within-subjects factor. The VSTMB was first analyzed using a 2×3 ANOVA with Group (LBC vs HBC) as the between-subjects factor and Condition (i.e., Shape Only vs Color Only vs Shape-Color Binding) as the within-subjects factor. Bonferroni-corrected post-hoc tests helped unveil the source of main effects and the interaction. The aim of this analysis was to investigate if the previously reported interaction denoting a specific feature binding impairment was present and if so, whether this was solely driven by a deficit in feature binding. Hence, our interest was on the single feature conditions as the Shape-Color Binding condition drove the group classification (i.e., to avoid circularity). For the analysis of neuropsychological data, we relied on False Discovery Rate (FDR (33)) corrected independent-sample t-tests. All the results we report were corrected for multiple comparisons.

##### Neuroimaging data

Amyloid SUVR were tested in regions of interest (ROI) and voxelwise. In ROIs, mean normalized amyloid SUVRs were obtained from 68 regions. We selected a subset of ROIs following three motivations. First, we were particularly interested in exploring areas of the brain known to display AD pathology in Braak stages I-III (34) which others have suggested to be accessible via context-free memory tests such as the VSTMBT (3, 7). Second, we combined such clinicopathological evidence with evidence drawn from earlier studies in clinical sample showing association between Aβ and VSTMB performance (6, 13). Finally, we want to improve our understanding of the precise neural correlates of memory decline in the preclinical stages of AD. By contributing domain-specific theory-driven cognitive tests we will refine our understanding of the core cognitive changes that characterize the transition from normal ageing to AD (35, 36). We therefore selected regions from frontal (37), parietal (38–40), occipital (38, 41), and temporal lobes (2, 7, 18, 42, 43). PET regional uptake values entered between-group contrasts (i.e., LBC vs HBC) across brain regions. Volumetric and cortical thickness data were also contrasted across groups (LBC vs HBC). Voxelwise, we explored correlations between amyloid SUVR and binding cost across participants, restricted to voxels with at least 50% probability of being gray matter. The ROI analyses (i.e., between group contrasts via independent-sample t-tests, correlation, whole-brain voxelwise analysis) were corrected for lobar volume and multiple comparisons (False Discovery Rate, FDR (33)).

## Results

### Behavioral outcomes

#### VSTMB

After applying the cut-off for the Cost of Binding, 21 asymptomatic older adults were classified as LBC and 18 were classified as HBC (see Supplementary Table 2 for demographic information from the current sample and the larger sample from which this was recruited). A mixed ANOVA model was used to test if our classification of participants in LBC vs HBC would yield a Group × Condition interaction confirming that binding impairments observed in the latter group would be accompanied by preserved memory for individual features. Although the effect of Group failed to reach significance [F(1,39) = 1.93; p = 0.173; η2= 0.05; β=0.23] the Group × Condition Interaction was significant [F(2,78) = 24.88; p < 0.001; η2= 0.39; β=1.0]. Bonferroni corrected post-hoc tests confirmed that neither Shape Only nor Color Only yielded significant differences between LBC and HBC [t(39) = 0.92, p = 0.363 and t(39) = 1.24, p = 0.225, respectively. Finally, relative to HBC, LBC had a numerically though not statistically higher level of Education [16.55±2.02 vs 15.45±1.79, p = 0.071], Premorbid IQ (WTAR, [41.11±7.19 vs 38.53±9.55, p = 0.36]) and Vocabulary (WAIS-III Vocabulary subtest [58.21±8.05 vs 57.28±9.43 p = 0.77]). We therefore decided to run the above model controlling for the effects of these variables. That did not remove the key Group × Condition Interaction [F(2,78) = 12.57; p < 0.001; η2= 0.32; β=0.98].

#### Traditional neuropsychological tests

LBC and HBC groups could not be distinguished based on their performance on standard neuropsychological tests (see Figure 3). The graph shows uncorrected p-values (blue diamond) and corrected q-values (orange square) for each test.

#### Aβ and VSTM

ROIs where Aβ deposits were significantly greater in HBC relative to LBL were mainly located in posterior regions of the brain. Bilateral involvement was found in the superior parietal lobe, superior and middle temporal gyrus, pericalcarine cortex and lingual gyrus. Interhemispheric discrepancies were found for the inferior parietal lobe (only left) and for the lateral-occipital cortex, entorhinal cortex, fusiform gyrus, and cuneus (only right) (Figure 4.A).

**Figure 2.**
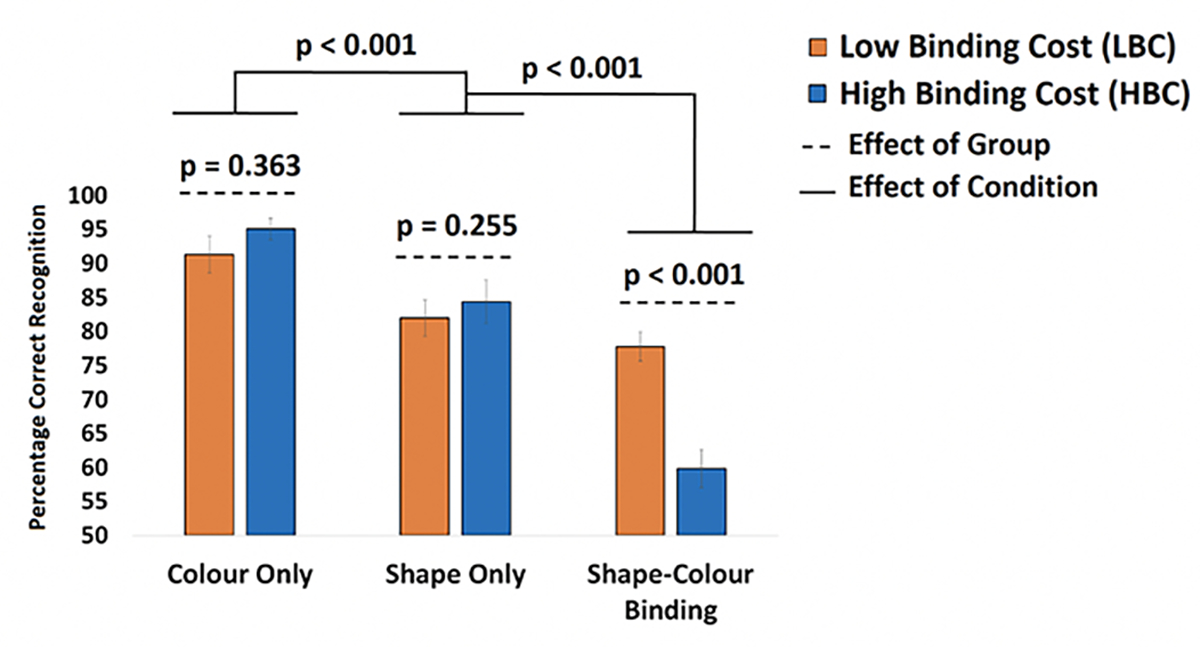
Mean performance from LBC and HBC across the three conditions of the VSTMB Task

**Figure 3.**
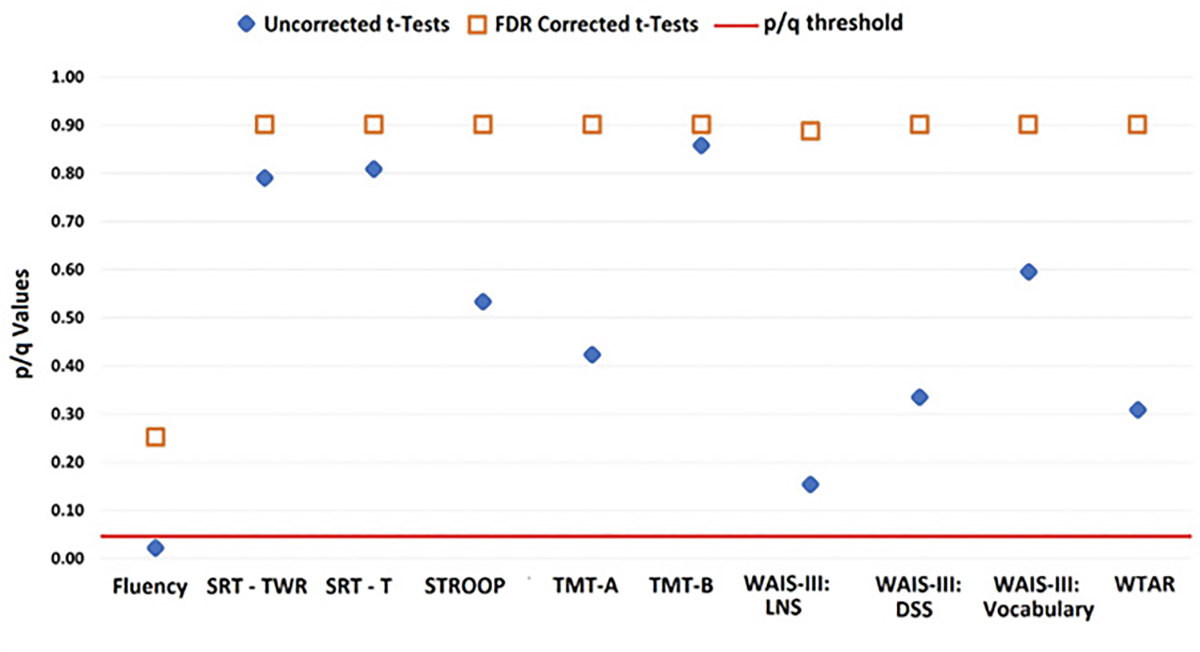
Data from LBC and HBC groups on an extensive neuropsychological test battery

**Figure 4.**
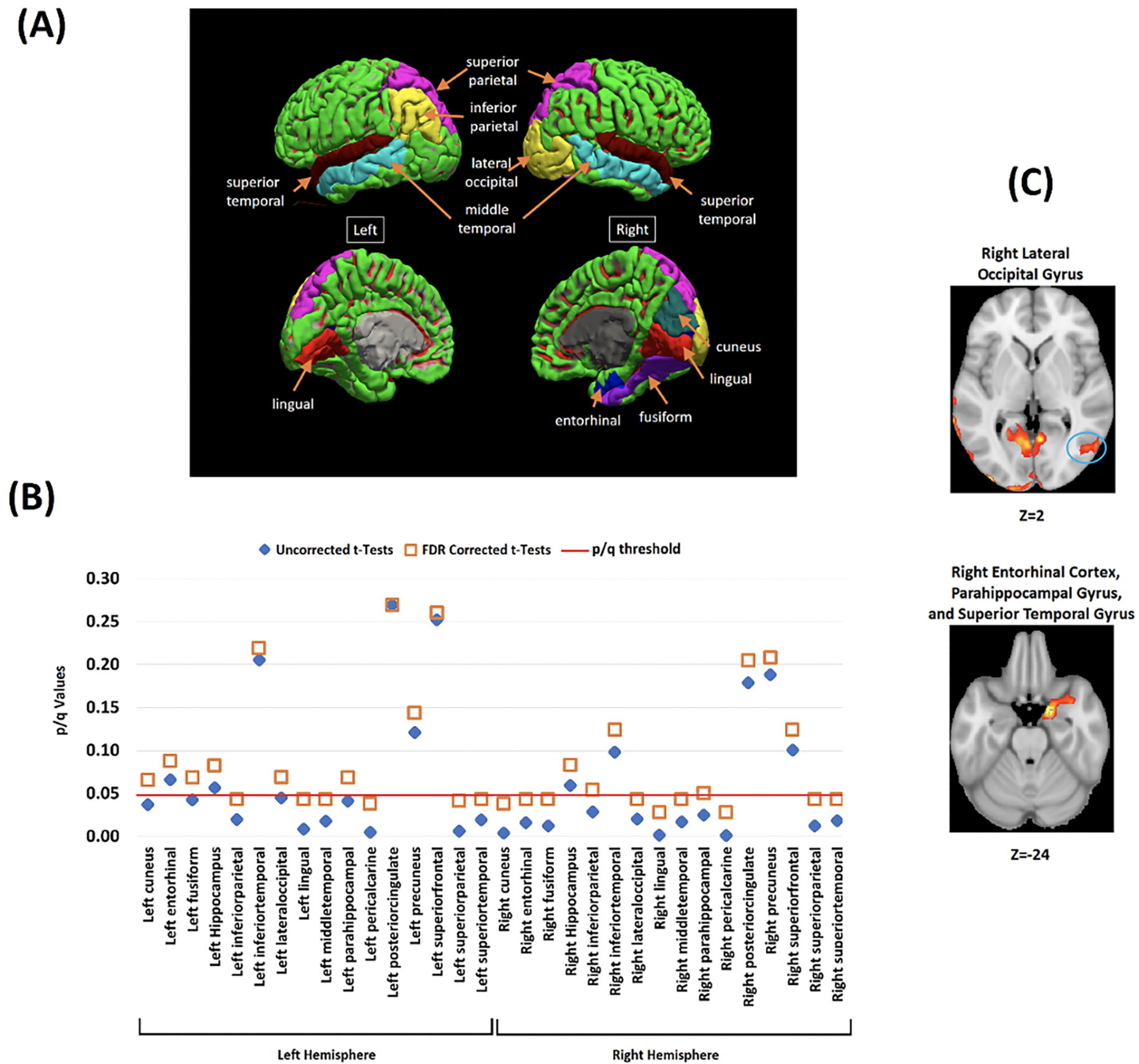
Analysis of the association between VSTMB and Amyloid-β in the targeted ROI

Figure 4.B shows that group discrepancies in these regions survived FDR correction (see Table 3 and Figure 1 in Supplementary Material for further regions of interest and correlation analyses). Voxelwise correlation analyses revealed significant positive correlations between Aβ deposits and the cost of binding in all the regions where between-group discrepancies were found (Figure 4.B, see also Figure 2 and Table 4 and in Supplementary Material).

#### Volume, Cortical Thickness and VSTMB

**Figure 5.**
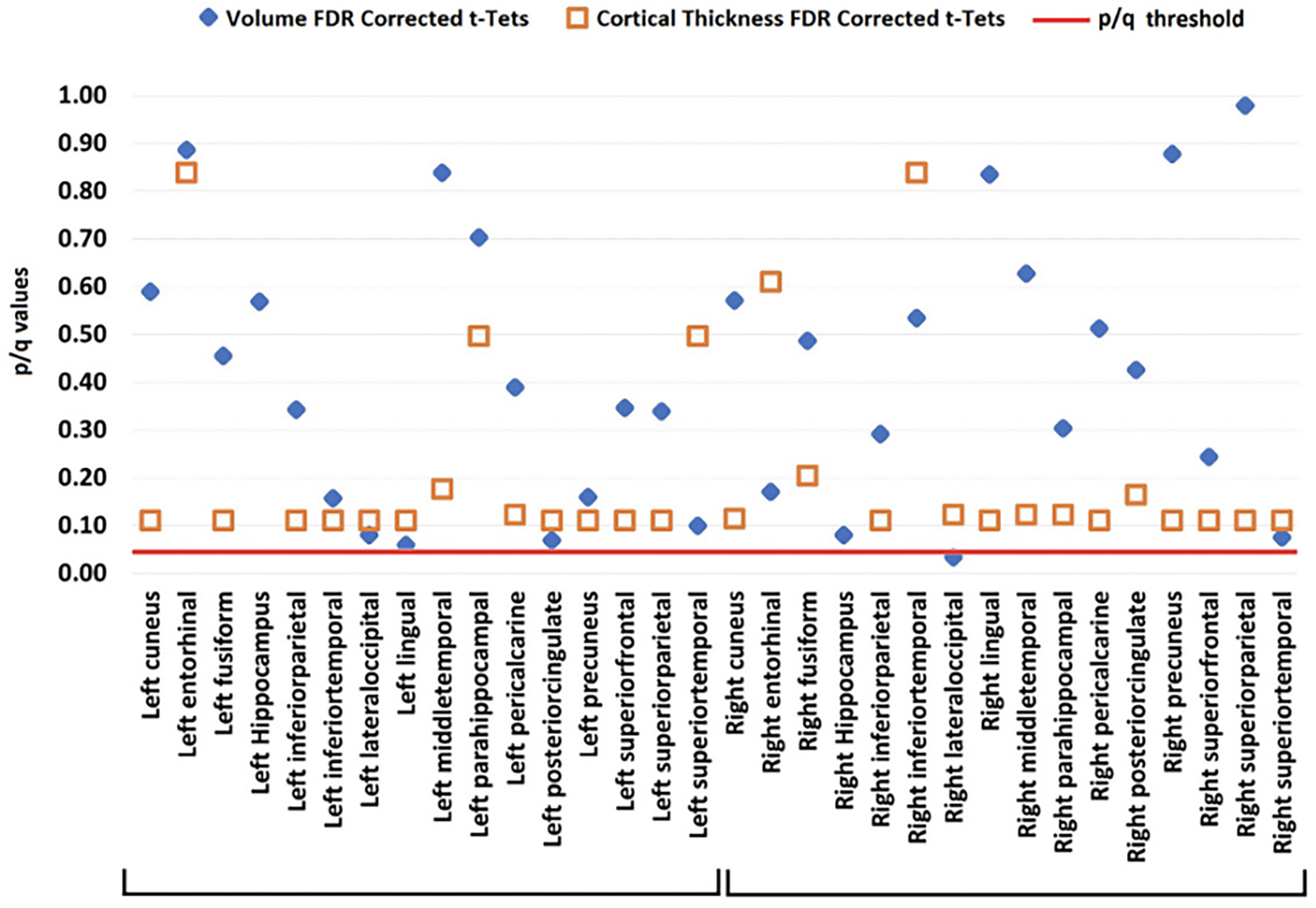
Volume (blue diamond) and cortical thickness (orange square) from LBC and HBC groups across the investigated brain regions (all corrected for multiple comparisons)

Neither volumetric nor cortical thickness measures differed between LBC and HBC. Only the right lateral-occipital cortex reached marginal FDR corrected values (see Figure 5).

## Discussion

The present study was set out to investigate the hypothesis that cognitively unimpaired older adults who present with VSTMB impairments would display increased brain Aβ deposits relative to those whose VSTMB remain preserved. This hypothesis proved valid. We also found that such an association occurred when neither measures of grey matter integrity nor standard neuropsychological tests could identify differences between these groups. These findings have important implications for our understanding of the boundaries between normal and pathological cognitive aging and for the preclinical detection of Alzheimer’s disease. We discuss such implications in turn.

VSTMB has been found to remain preserved across the lifespan (11, 12, 44) and to be unaffected by the level of education of those assessed (45). The hypothesis that VSTMB impairments in asymptomatic older adults would reflect early Aβ pathology stemmed from recent studies in individuals at high risk of AD. VSTMB impairments have been found in middle-age adults who would inevitably develop familial AD due to the mutation E280A-PSEN1 (10) and who were otherwise completely asymptomatic. Aβ deposits in such carriers reach a plateau at the mean age of 35 (46), which is when VSTMB impairments were first observed (10). The association between Aβ and VSTMB impairments in asymptomatic carriers of the mutation E280A-PSEN1 becomes apparent before evidence of tau pathology or neurodegeneration (6). Interestingly, such an association also characterizes individuals at risk of late-onset sporadic AD (i.e., mild cognitive impairment) (13). The still scarce yet converging evidence suggests that VSTMB deficits might be associated to the earliest pathological changes that underpin the transition from normal aging to AD, that is, β-amyloidopathy.

Disentangling normal and pathological cognitive aging is a challenge that neuropsychological tests are currently facing (20). There is growing concern about the reliability of norms or control groups as the available neuropsychological tests currently used to ascertain normality are outdated and do not detect the earliest cognitive deficits caused by neurodegenerative disease. The traditional neuropsychological tests used in our study proved insensitive to the increased Aβ observed in older adults with poor VSTMB functions. In fact, participants enrolled in this study were recruited relying on strict inclusion criteria for normal cognitive aging. Yet, almost half of them presented with a behavioral VSTMB profile compatible with that consistently observed in individuals with or at risk of AD (10, 47, 48) (Figure 2). In fact, the performance of the HBC group on the baseline conditions was numerically superior to that of the LBC group, which our manipulation (i.e., classification into LBC and HBC) would not predict. That is, our manipulation did anticipate that relative to LBC, HBC would show significantly poorer performance on the Shape-Color Binding Condition. This is the dissociation previously observed in people with or at risk of AD (10, 30, 31) and our data confirmed this prediction (see Figure 2). However, such manipulation would warrant neither equivalent performance on single feature conditions nor a significant Group × Condition Interaction. These findings therefore grant us confidence that HBC did present with the typical binding profile previously identified in population with or at risk of AD dementia.

It is worth noting that our neuropsychological assessment battery included the Selective Reminding Test, which has been considered a preclinical cognitive marker for AD (1). As we highlighted in the Introduction, these two forms of memory binding have proved dissociable in patients with AD or at risk of this type of dementia. The differential sensitivity of the forms of memory binding assessed by the VSTMBT and SRT to the transition from normal aging to AD has been recently noted (20). The observation that VSTMB impairments were associated to increased deposits of Aβ in brain regions known to support visual object processing and memory contributes novel insights into the earliest neurocognitive changes that will likely characterize such a transition. We discuss such links next.

(A) The investigated brain regions drawn from whole-brain voxelwise analysis. (B) Between-groups contrasts for Aβ SUVRs in HBC relative to LBC across the investigated brain regions (Blue diamond = Uncorrected tests, Orange Square = FDR correction for multiple comparisons). The Y axis shows the uncorrected and corrected (q) p-values. (C) Whole-brain voxel-wise analyses (between-groups) illustrating two key brain regions where Aβ deposits significantly correlated with the cost of binding (all corrected for multiple comparisons) (see also Supplementary Figure 1 and 2).

VSTMB appears to be linked to the functions of the visual ventral stream (49). Cortico-cortical connections along this pathway support object unitization and identity formation. However, the occipitotemporo-medialtemporal pathway plays a key role in memory (20, 49). This pathway consists of projections from the cortical components to various structures within the medial temporal lobe including the perirhinal cortex, which projects in turn to both the entorhinal cortex and to regions of the hippocampus. These regions of the anterior temporal network are thought to support familiarity-based recognition, a function known to support performance on change detection tasks such as the VSTMBT. VSTMB remains preserved in patients with hippocampal damage (9), and is affected in patients at risk of AD who still perform normally on memory tests that tax the function of the hippocampus (10, 30, 50). This suggests that pathology during the transentorhinal stage of AD, which appears prior to the hippocampal stage (3), might be the one the VSTMBT is detecting. Accrued evidence seems to support this notion. While the hippocampus undergoes substantial atrophy as we grow older, the volume of the perirhinal and entorhinal appears to be unaffected by age (5). Interestingly, these regions are targeted by AD before the hippocampus (4, 51). This would explain why VSTMB has been consistently found to be insensitive to normal aging and sensitive to AD in its subhippocampal transentorhinal stage.

Evidence gathered to date suggests that the above sequence of neuropathological events is seemingly driven by tau pathology i.e., deposits of neuro-fibrillary tangles (NFT) in regions of the anterior network of the medial temporal lobe. In fact, a hypothetical model that maps the earliest memory impairments detectable in AD to the underlying neuropathology (3) suggests that NFT in the sub-hippocampal stages of AD may account for the type of deficit we find with the VSTMBT (context-free memory impairments). Our data suggests that increased Aβ in the same regions of such a network can also disrupt such a memory function. Taking together the evidence above reviewed and that drawn from our own study we feel compelled to suggest that the VSTMBT appears to be indexing Aβ pathology in the very early stages of the AD continuum, seemingly before tau pathology becomes apparent. Studies using animal models have confirmed that tau is not necessary for Aβ to induce memory impairments (52). In fact, tau pathology in humans seems to account for stages where the abnormal brain structure–function relationships become detectable. Accrued wisdom suggests that this may be too late when it comes to dementia prevention.

One may question whether the dichotomization approach used in this study is a reliable methodology to explore the association of cognition and AD pathology (both moving along a continuum) in cognitively unimpaired older adults. While dichotomization is often necessary and clinically useful, it often carries some challenges (see for example Morris (53)) regarding the impact of cut-off scores of functional scales on MCI/AD diagnosis). Notwithstanding such challenges, Forno et al. (21) recently confirmed that such a methodological approach allowed them to identify subtle cognitive impairments in people at risk of AD who had been otherwise undetected. In the context of the present study, we observed at a whole-group level analysis (see Supplementary Figure 1 and 2, correlations and whole-brain voxel-wise analyses) that increased Aβ in ROIs known to be nodes of the VSTMB network significantly predicted increase in the memory binding cost. It is worth noting that classical procedures to classify participants based on Aβ -PET would have faced limitations in the current sample as our cognitively unimpaired older adults were largely subthreshold (see (6) for evidence from clinical samples). Taken together these findings suggest that our approach to dichotomize the sample should not be a factor undermining the reliability of our results.

The findings here reported come from a relatively small cross-sectional sample of healthy older adults. Future studies will be needed to validate these results in larger longitudinal samples. Lending support to this suggestion, Parra et al. (19) recently observed that the VSTMBT is a reliable predictor of progression from normal to pathological aging, as defined by the very early stages of MCI. The authors suggested that it is at this stage when the test stands the best chance to identify those will progress to AD dementia (20). Taken together these and our results suggest that the novel memory marker here investigated could help identify those presymptomatic older adults who are currently missed by available cognitive screening procedures.

In fact, evidence from CSF/PET Amyloid findings in asymptomatic adults suggests that preclinical and prodromal AD may be more prevalent than previously estimated (54). This might have important implications for clinical trial recruitment strategies and for the development of normative samples. Regarding the latter, some have already suggested the need of biomarker adjusted normative data to reliably separate normal and pathological aging trajectories (55). An alternative would be to rely on theory-driven function-specific cognitive tests capable of unveiling the earliest manifestation of AD. The VSTMBT seems to be a promising candidate. Dementia prevention entails both early detection and effective treatments, and both are currently lacking (20). The results here presented grant us confidence to suggest that the VSTMBT can be considered a promising screening tool to help identify individuals who can be good candidates for AD prevention trials.

## Supplementary Material

supplementary material
