# Supplementary material for: Exploring the Association between Amyloid-β and Memory Markers for Alzheimer’s Disease in Cognitively Unimpaired Older Adults
Source: J Prev Alzheimers Dis. Author manuscript; Available in PMC 2024 Apr 11. (PMC11007669; doi:10.14283/jpad.2024.11)
Supplement: supplementary material [file NIHMS1979105-supplement-supplementary_material.docx]

**Exploring the association between amyloid-β and memory markers for Alzheimer’s disease in cognitively unimpaired older adults**

**Table 1.** Neuropsychological Tests included in the battery of tests used in the Cognitive Reserve Study^1^ and tests selected for the present study (see manuscript for motivation of such a selection).

| **Full Neuropsychological Battery** | | **Tests Selected for the Study** |
| --- | --- | --- |
| **Dementia Screen and General Intellectual functioning** | | |
| Mattis Dementia Rating Scale (DRS) | ✓ | |
| *Premorbid IQ* Wechsler Test of Adult Reading (WTAR) | ✓ | |
| Wechsler Adult Intelligence Scale – Third Edition (WAIS-III) – Vocabulary subtest | ✓ | |
| **Learning and Memory** | | |
| Selective Reminding Test | ✓ | |
| Wechsler Memory Scale-III |  | |
| Verbal Paired Associates |  | |
| **Basic Attention** | | |
| WAIS-III Digit Span forward |  | |
| Processing Speed |  | |
| Trail Making Test Part A | ✓ | |
| WAIS-III Digit Symbol subtest | ✓ | |
| Stroop Color Reading | ✓ | |
| **Executive functions** | | |
| a. *Inhibition* |  | |
| Stroop Color and Word Test | ✓ | |
| b. *Shifting* Wisconsin Card Sorting Test |  | |
| WCST |  | |
| Trail Making Test Part B; | ✓ | |
| c. *Revision & Monitoring* |  | |
| WAIS-III Letter-Number Sequencing | ✓ | |
| WAIS-III Digit Span backwards |  | |
| **Fluid Reasoning** | | |
| WAIS-III Similarities subtest |  | |
| WAIS-III Matrix Reasoning subtest |  | |
| **Reaction Time** | | |
| Variable-foreperiod simple reaction time |  | |
| Choice reaction time (go-no go task, 2-choice RT and 4-choice RT) |  | |
| **Language** | | |
| Boston Naming Test |  | |
| Controlled Oral Word Association Test | ✓ | |
| Category Fluency Test | ✓ | |
| **Visuoconstruction** | | |
| WAIS-III Block Design subtest |  | |

1. Habeck C, Gazes Y, Stern Y. Age-Specific Activation Patterns and Inter-Subject Similarity During Verbal Working Memory Maintenance and Cognitive Reserve. Original Research. *Frontiers in Psychology*. 2022-June-09 2022;13doi:10.3389/fpsyg.2022.852995

**Table 2.** Demographic information for the participants that agreed to participate in the study and those that received amyloid PET but declined participate in the current study. There was no statistical difference between the two groups in these demographic information.

|  | **In study (N=39)** | **Not in study (N=62)** | **p-value** |
| --- | --- | --- | --- |
| Age - Mean(SD) | 65.3 (3.07) | 65.3 (3.33) | 0.793 |
| Sex - F/M | 16/23 | 35/27 | 0.131 |
| Education - Mean(SD) | 16.2 (1.90) | 16.3 (2.35) | 0.861 |
| WTAR - Mean(SD) | 111 (13.1) | 111 (13.1) | 0.972 |

**WTAR:** Wechsler Test of Adult Reading.

**Table 3.** Brain regions revealing significant differences in Aβ deposits between LBC and HBC during whole-brain voxelwise analysis.

| **Voxels** | **Max lodP** | **X (mm)** | **Y (mm)** | **Z (mm)** | **Brain regions** |
| --- | --- | --- | --- | --- | --- |
| 10347 | 4.38 | -24 | -63 | -4 | Bilateral lingual and fusiform gyri |
| 5871 | 3.56 | -8 | -93 | 10 | Bilateral cuneus and supracalcarine |
| 5028 | 2.99 | 65 | -51 | 20 | Right angular and middle temporal gyri, right lateral occipital cortex |
| 3776 | 2.94 | -51 | -79 | 11 | Left middle temporal gyrus and lateral occipital cortex |
| 1787 | 3.23 | 17 | 1 | -24 | Right entorhinal cortex, parahippocampal gyrus, and temporal pole |
| 1123 | 2.41 | -64 | -45 | 15 | Left superior and middle temporal gyri, and angular and supramarginal gyri |
| 1015 | 1.89 | 44 | -31 | 54 | Right postcentral gyrus and superior parietal lobule |
| 961 | 3.04 | -37 | -95 | 2 | Left lateral occipital cortex and occipital pole |
| 718 | 2.37 | -45 | 6 | 30 | Left middle and inferior frontal gyri, and precentral gyrus |
| 587 | 2.25 | 34 | -78 | 24 | Right lateral occipital cortex |

**Figure 1.** Scatterplots depicting the correlations between the Binding Cost and the accumulation of Amyloid-β in four ROI known to be nodes of the VSTMB network: **(A)** right cuneus, **(B)** right fusiform gyrus, **(C)** right lateral occipital cortex, and **(D)** right entorhinal cortex. We show correlations that survived FDR corrections.

**
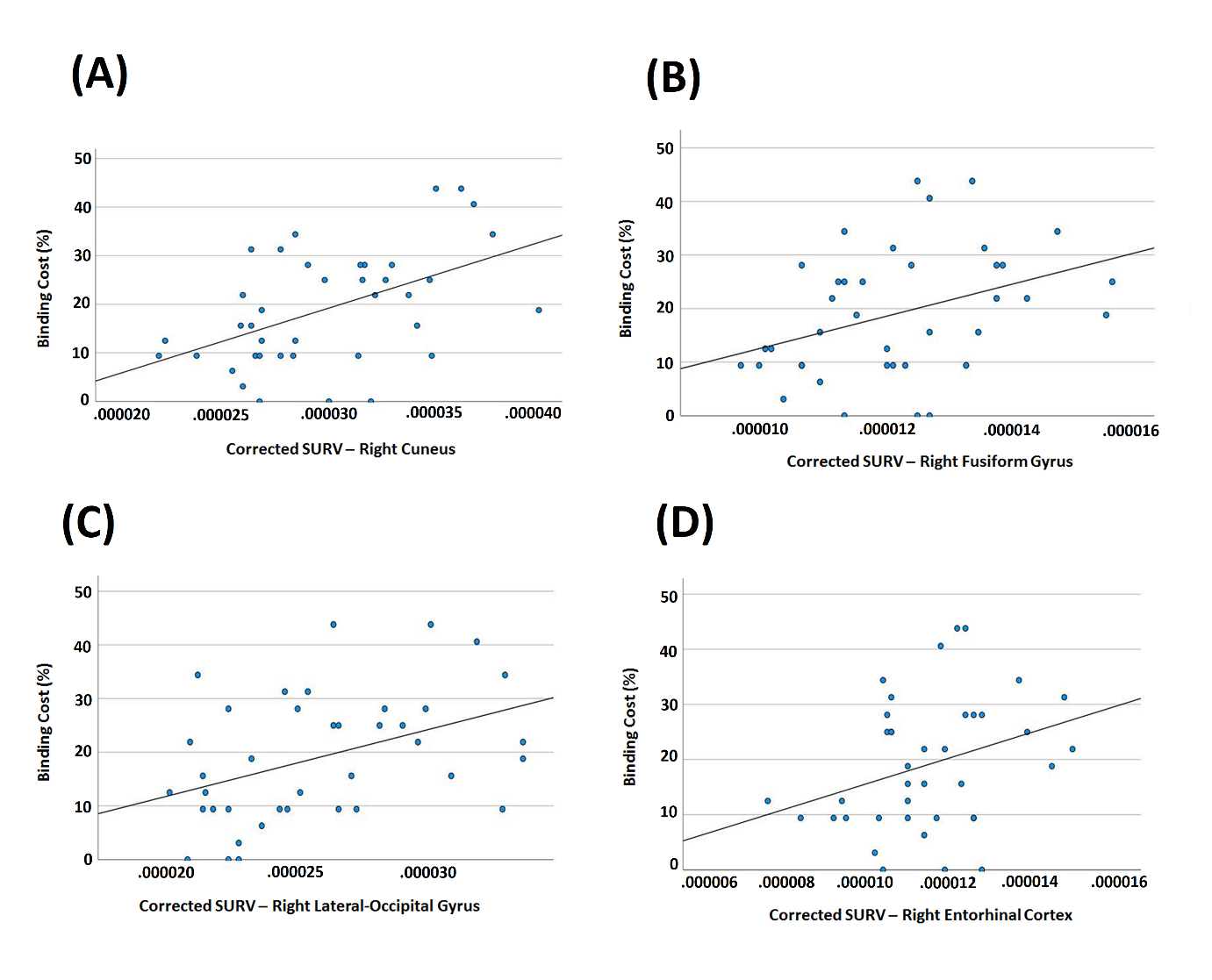
**

Figure 2. Additional ROI where whole-brain voxel-wise analyses (between-groups) revealed that Aβ deposits significantly correlated with the cost of binding (all corrected for multiple comparisons) (see also Figure 5 in main manuscript).

**
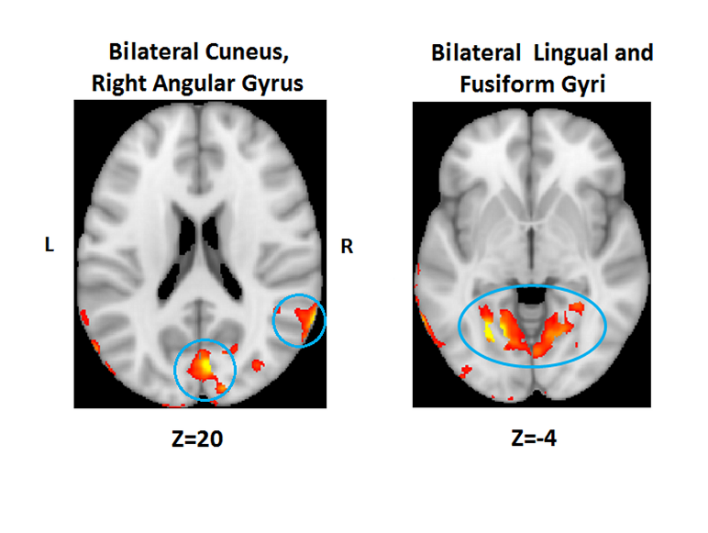
**

**Table 4.** Results from the correlation analysis. We show correlations that survived FDR corrections (see Figure 4.A in manuscript for the full set of correlations).

| **Correlations with Binding Cost** | **Statistic** | | |
| --- | --- | --- | --- |
|  | ***r*** | **p-value** | **CI** |
| Left superior temporal gyrus | 0.366 | 0.022 | (0.057-0.611) |
| Right superior temporal gyrus | 0.369 | 0.021 | (0.061-0.613) |
| Left middle temporal gyrus | 0.352 | 0.028 | (0.041-0.601) |
| Right middle temporal gyrus | 0.383 | 0.016 | (0.077-0.623) |
| Left superior parietal lobule | 0.375 | 0.019 | (0.068-0.618) |
| Right superior parietal lobule | 0.355 | 0.027 | (0.044-0.603) |
| Left pericalcarine cortex | 0.522 | 0.001 | (0.247-0.719) |
| Right pericalcarine cortex | 0.565 | 0.000 | (0.304-0.747) |
| Left lingual gyrus | 0.473 | 0.002 | (0.185-0.686) |
| Right lingual gyrus | 0.534 | 0.000 | (0.262-0.727) |
| Left inferior parietal lobule | 0.407 | 0.010 | (0.105-0.641) |
| Right lateral occipital cortex | 0.419 | 0.008 | (0.119-0.649) |
| Right entorhinal cortex | 0.321 | 0.046 | (0.006-0.578) |
| Right fusiform gyrus | 0.379 | 0.017 | (0.072-0.620) |
| Left cuneus | 0.453 | 0.004 | (0.161-0.672) |
